# First record of *Neoempheria* Osten Sacken (Diptera, Mycetophilidae) biology in the Neotropical region, with associations between its larvae and fungi

**DOI:** 10.3897/BDJ.3.e5073

**Published:** 2015-06-08

**Authors:** Sarah Siqueira Oliveira, Fabiano Fabian Albertoni, Christopher James Borkent, Dalton S. Amorim

**Affiliations:** ‡ Departamento de Ecologia, Instituto de Ciências Biológicas, Universidade Federal de Goiás (UFG), Goiânia, Brazil; § MZSP, São Paulo, Brazil; | California State Collection of Arthropods, Sacramento, United States of America; ¶ DB/ FFCLRP/USP, Ribeirão Preto, Brazil

**Keywords:** Immature, Fungivore, Natural History, Biodiversity, Ecology

## Abstract

**Background:**

Members of the family Mycetophilidae (Diptera) have life cycles that are typically associated with fungus. Their biology is relatively well known in the Palaearctic, though other regions are poorly known, and there are no associations recorded between mycetophilid immatures and fungi in the Neotropical region. Here we report the first association between a mycetophilid—*Neoempheria puncticoxa* Edwards—and fungi in this region. Immatures of *N. puncticoxa* were collected on fungi and some were reared in the laboratory until adult emergence. The immature stages and adult of *N. puncticoxa* are described and re-described respectively, and high resolution images and illustrations of the habitus, wings, thorax, male and female terminalia, immatures, and *in situ* specimens are given.

**New information:**

We report the first association between Mycetophilidae and fungi in the Neotropical region.

## Introduction

Mycetophilidae (Diptera) is composed of 233 genera and ~4500 described species from all biogeographic regions (Pape et al. 2011). In the Neotropical region it is one of the most species-rich dipteran families with 1145 described species (Oliveira and Amorim 2014).

Mycetophilidae adults are often found in moist environments—usually in damp woods, tunnels, in the cavities under tree roots, and under stream bank overhangs (Hutson et al. 1980, Ševčík 2010). All known mycetophilid larvae feed on a component of fungi or myxomycetes (mycelium, fruiting bodies and spores), but the biology of most species still remains unknown (Hutson et al. 1980, Ševčík 2010).

A number of papers have been published on the biology of mycetophilids, indicating their association with fungi in the Palearctic and Nearctic (*e.g.*, Hutson et al. 1980, Kurina 1994, Ševčík 2010, Jakovlev 2011, Borkent and Wheeler 2012). Unfortunately their role in forest ecosystems as fungivores remains virtually unexplored (but see Põldmaa et al. 2015). The most comprehensive review on the morphology of immature Mycetophilidae *s.l.* is the series of papers by Plachter (Plachter 1979b, Plachter 1979a, Plachter 1979c), though a number of important earlier contributions should be noted (Osten Sacken 1862, Madwar 1937, Buxton 1960). Information on the biology of *Leptomorphus* has also been recently studied (Eberhard 1990, Borkent and Wheeler 2012). However, nothing has been reported on the larval biology of Neotropical species of Mycetophilidae. Here we provide the first observations on the larvae of a Neotropical species of mycetophilid, *Neoempheria puncticoxa* Edwards and its fungal associations, along with morphological descriptions of the different life stages.

## Materials and methods

Samples of fungi were collected in two different areas of Brazil, one in the State of Goiás (by FFA) and another in the northeast part of the State of São Paulo (by CJB and SSO). Both these areas have a biome classified as Semi-deciduous Forest, with a dry season from June to September. The fungi samples with mycetophilid larvae were transferred to plastic jars covered by a fine mesh. Samples were misted daily with water to maintain humidity. Five adults emerged from the fungi. All material examined, with the exception of the holotype, was kept pinned, preserved in 80% alcohol, or on slides, and housed in the Diptera collection of the Museu de Zoologia da Universidade de São Paulo (MZSP), São Paulo, Brazil. The holotype of *N. puncticoxa* was examined at the Natural History Museum (NHM), London, UK (additional information on the holotype is available in Amorim and Oliveira 2013).

Methods for the preparation of specimens, photos and illustrations follow Oliveira and Amorim (2012). Morphological terminology follows Søli (1997) and Amorim and Rindal (2007) for adults and Courtney et al. (2000) for larvae. Field photos were taken with either a Canon EOS 5D Mark II camera with a Canon EF 100mm F2.8 *L* IS USM Macro lens and Canon Speedlite 580EX II Flash (FFA), or a Canon PowerShot SX200IS (CJB).

## Data resources

### Material examined

***Neoempheria puncticoxa*** Edwards

**Immatures reared.** 5 larvae, BRAZIL, *Goiás*, Goiânia, Polyporales fungi collected 22.i.2012, which 2 larvae pupated 05.ii.2012, 2♂ emerged 09.ii.2012, F.F. Albertoni Leg., det. S.S. Oliveira, viii.2013; 3♀, BRAZIL, *São Paulo*, Ribeirão Preto, Campus of the University of São Paulo, 21°13'30"S 47°51'01"W, *Sparassis* fungi collected on 19.iii.2013, adults emerged 05-09.iv.2013, S.S. Oliveira & C.J. Borkent Leg., det. S.S. Oliveira, viii.2013.

**Additional adult material examined.** *N. puncticoxa* Edwards holotype ♂ (see Amorim and Oliveira 2013); 1♂ BRAZIL, *Mato Grosso*, Nova Mutum, Fazenda Buriti, 17.i.2000, H.F. Mended Leg., det. Oliveira, viii.2013; 1♂ BRAZIL, São Paulo, Santo Amaro, iii.1949, John Lane Leg., det. E. Coher 1952; 1♂ (terminalia lost), BRAZIL, São Paulo, São José dos Campos, i.1937, H.S. Lopes Leg., E. Coher det. 1952; 1♂, BRAZIL, *Mato Grosso do Sul*, Maracajú, vi.1937, Serviço Febre Amarela M.E.S., Brasil, R.C. Shannon Collection, J. Lane det. 1948; E. Coher det. 1952; 1♀, BRAZIL, Goiás, Corumbá de Goiás, xi.1945, Barretto Leg, J. Lane det. 1948.

## Taxon treatments

### Neoempheria puncticoxa

Edwards, 1940

Neoempheria Osten Sacken 1878: 9 (*nom. nov.* for *Empheria* Winnertz). Type-species, *Sciophila striata* Meigen (aut.).
Neoempheria puncticoxa *Neoempheria puncticoxa*Edwards 1940: 115, fig. 5 (♂ terminalia). Type locality: Brazil, Santa Catarina, Seara, Nova Teutônia. Distr.: Brazil (Santa Catarina, São Paulo, Mato Grosso, Mato Grosso do Sul, Goiás), Argentina (Salta, Tucuman). Refs.: Coher 1959: 24; Amorim and Oliveira 2013: 68 (type comments and label data), fig. 193 (habitus). Holotype ♂, NHM.
Neoempheria puncticoxa (Figs 1, 2, 3, 4, 5, 6, 7, 8, 9, 11, 12, 13, 14, 15)

#### Materials

- **Type status:** Other material. **Occurrence:** recordedBy: S.S. Oliveira & C.J. Borkent; individualCount: 1; sex: male; lifeStage: adult; **Taxon:** scientificName: Neoempheria puncticoxa; **Location:** country: Brazil; stateProvince: São Paulo; locality: Ribeirão Preto, Campus of the University of São Paulo; locationRemarks: Sparassis fungi collected on 19.iii.2013, adults emerged 05-09.iv.2013; decimalLatitude: -21.225; decimalLongitude: -47.85; **Identification:** identifiedBy: Sarah S. Oliveira; dateIdentified: 2013; **Event:** samplingProtocol: Reared; eventDate: 03/19/2013; **Record Level:** collectionID: http://grbio.org/cool/9yp6-zxp9; collectionCode: MZSP; basisOfRecord: PreservedSpecimen
- **Type status:** Other material. **Occurrence:** recordedBy: F.F. Albertoni; individualCount: 2; sex: male; lifeStage: adult; **Taxon:** scientificName: Neoempheria puncticoxa; **Location:** country: Brazil; stateProvince: Goiás; locality: Goiânia; locationRemarks: 3 larvae and a Polyporales fungi collected 22.i.2012;, larvae pupated 05.ii.2012, 2♂ emerged 09.ii.2012; decimalLatitude: -16.68; decimalLongitude: -49.26; **Identification:** identifiedBy: Sarah S. Oliveira; dateIdentified: 2013; **Event:** samplingProtocol: Reared; eventDate: 02/09/2012; **Record Level:** collectionID: http://grbio.org/cool/9yp6-zxp9; collectionCode: MZSP; basisOfRecord: PreservedSpecimen
- **Type status:** Holotype. **Occurrence:** catalogNumber: BMNH(E)257767; recordedBy: Friedrich 'Fritz' Plaumann; individualCount: 1; sex: male; lifeStage: adult; **Taxon:** scientificName: Neoempheria puncticoxa; **Location:** country: Brazil; stateProvince: Santa Catarina; locality: Nova Teutonia; decimalLatitude: -27.18; decimalLongitude: -52.38; **Event:** eventDate: 04/21/1938; **Record Level:** collectionID: http://biocol.org/urn:lsid:biocol.org:col:34665; collectionCode: BMNH; basisOfRecord: PreservedSpecimen
- **Type status:** Other material. **Occurrence:** recordedBy: John Lane; individualCount: 1; sex: male; lifeStage: adult; **Taxon:** scientificName: Neoempheria puncticoxa; **Location:** country: Brazil; stateProvince: São Paulo; locality: Santo Amaro; decimalLatitude: -23.65; decimalLongitude: -46.71; **Identification:** identifiedBy: E. Coher; dateIdentified: 1952; **Event:** eventDate: 1949-3; **Record Level:** collectionID: http://grbio.org/cool/9yp6-zxp9; collectionCode: MZSP; basisOfRecord: PreservedSpecimen
- **Type status:** Other material. **Occurrence:** recordedBy: H.F. Mended; individualCount: 1; sex: male; lifeStage: adult; **Taxon:** scientificName: Neoempheria puncticoxa; **Location:** country: Brazil; stateProvince: Mato Grosso; locality: Nova Mutum, Fazenda Buriti; decimalLatitude: -15.64; decimalLongitude: -54.17; **Identification:** identifiedBy: Sarah S. Oliveira; dateIdentified: 2013; **Event:** eventDate: 01/17/2000; **Record Level:** collectionID: http://grbio.org/cool/9yp6-zxp9; collectionCode: MZSP; basisOfRecord: PreservedSpecimen
- **Type status:** Other material. **Occurrence:** recordedBy: H.S. Lopes; individualCount: 1; sex: male; lifeStage: adult; **Taxon:** scientificName: Neoempheria puncticoxa; **Location:** country: Brazil; stateProvince: São Paulo; locality: São José dos Campos; decimalLatitude: -23.22; decimalLongitude: -45.9; **Identification:** identifiedBy: E. Coher; dateIdentified: 1952; **Event:** eventDate: 00/1/1937; **Record Level:** collectionID: http://grbio.org/cool/9yp6-zxp9; collectionCode: MZSP; basisOfRecord: PreservedSpecimen
- **Type status:** Other material. **Occurrence:** recordedBy: Serviço Febre Amarela M.E.S., Brasil, R.C. Shannon Collection; individualCount: 1; sex: male; lifeStage: adult; **Taxon:** scientificName: Neoempheria puncticoxa; **Location:** country: Brazil; stateProvince: Mato Grosso do Sul; locality: Maracajú; decimalLatitude: -21.64; decimalLongitude: -55.16; **Identification:** identifiedBy: J. Lane (1948), E. Coher; dateIdentified: 1952; **Event:** eventDate: 00/6/1937; **Record Level:** collectionID: http://grbio.org/cool/9yp6-zxp9; collectionCode: MZSP; basisOfRecord: PreservedSpecimen
- **Type status:** Other material. **Occurrence:** recordedBy: Barretto; individualCount: 1; sex: female; lifeStage: adult; **Taxon:** scientificName: Neoempheria puncticoxa; **Location:** country: Brazil; stateProvince: Goiás; locality: Corumbá de Goiás; decimalLatitude: -15.93; decimalLongitude: -48.81; **Identification:** identifiedBy: J. Lane; dateIdentified: 1948; **Event:** eventDate: 00/9/1945; **Record Level:** collectionID: http://grbio.org/cool/9yp6-zxp9; collectionCode: MZSP; basisOfRecord: PreservedSpecimen

#### Description

**Redescription of adults**

**Male** (Figs 1, 3a, 5, 6, 14b): **Head** (Figs 1b, 5): Vertex brownish, with scattered setae, yellowish around eyes. Two ocelli medially on blackish vertex. Frons light brown. Face and clypeus yellowish, covered with setulae. Labellum yellowish, ventrally darker; maxillary palpus brownish, apical segment lighter, segments 3-5 of similar length, first two segments short. Scape and pedicel yellow, rounded, more setose anteriorly; flagellum brown, antenna shorter than thorax, flagellomeres slightly longer than wide. **Thorax** (Fig. 5): Prosternum brownish. Pronotum yellow, with strong, long, black bristles. Proepisternum yellow, proepimeron brownish posteriorly, both bare. Anepisternum yellow anteriorly with large brown macula on posterior two thirds, bare. Katepisternum yellow on dorsal third, large brown macula occupying ventral two thirds, bare. Mesepimeron yellow, posterior margin brownish, bare. Laterotergite mostly yellow, anterodorsal margin brownish, bare. Mediotergite yellow ventrally, a brownish triangular mark dorsally, bare. Meso- and metapleura yellow, entirely bare. Scutum yellow, with five brown stripes fusing posteriorly, covered with short and long setae, a pair of stronger dorsocentral and a pair of stronger dorsolateral setae posteriorly. Scutellum yellow, with a pair of long scutellar bristles and a few scattered setulae. Legs yellow; forecoxa with some brownish maculae anteriorly, with strong setae in a line on its posterior and ventral margins; tibial setae regularly aligned; tibial spur I almost twice length of tibial diameter at apex, tibial spurs II and III almost four times length of tibial diameter at apex. Halter stem whitish, knob black, setose. **Wing** (Fig. 3a): Wing venation and color pattern as in the Fig. 3a. **Abdomen** (Figs 1, 14b): Tergites and sternites mostly yellow; T1 brown posteromedially; T2 brown anteromedially; T3 brown, but yellow laterally; T4 and T6 brown medially, yellow posterolaterally; T5 brown with yellow posterior margin; T7 mostly yellow; sternites mostly yellow, sternites 3 and 5 with brown areas on its surface. **Terminalia** (Fig. 6): Yellow. T9 weakly developed and sclerotized, with a few setae distally (Fig. 6C). Gonocoxite with large dorsal projection extending beyond apex of gonostylus, densely covered with setae on external face, apex digitiform, mostly bare, a few small setae at apex. Gonostylus well developed, deeply bifid, inner branch secondarily bifid and nearly bare, a few small spines on inner margin, external branch strongly setose. Gonocoxal apodeme short, sclerotized; parameres strongly developed, with a dorsal, membranous, bare projection; cercus and S10 rounded, with some small setae distally (Fig. 6D).

**Figure 2.**
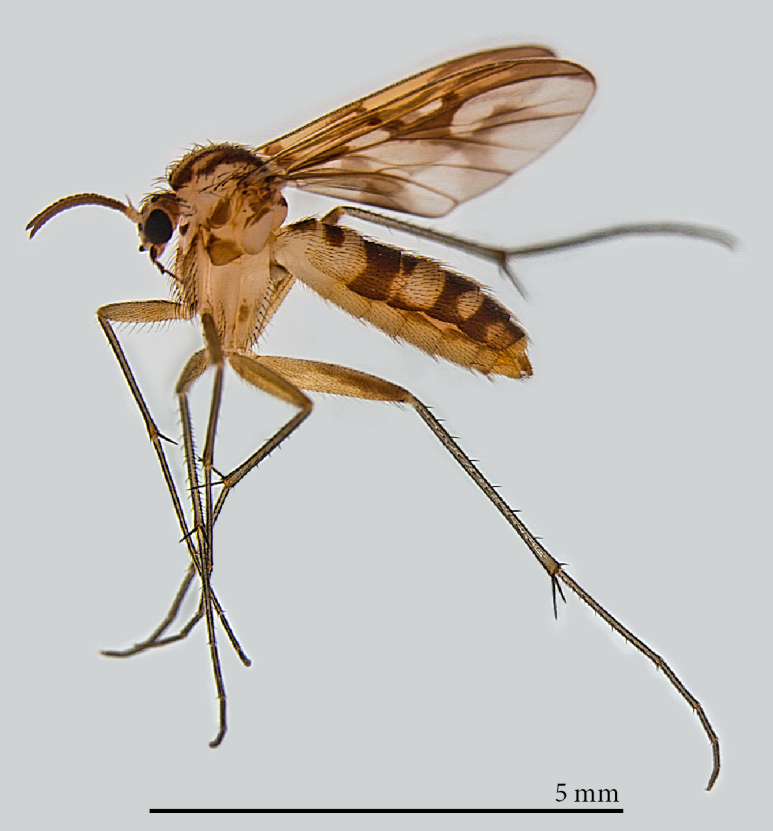
*Neoempheria puncticoxa* Edwards, 1940. Female adult, lateral view.

**Figure 4.**
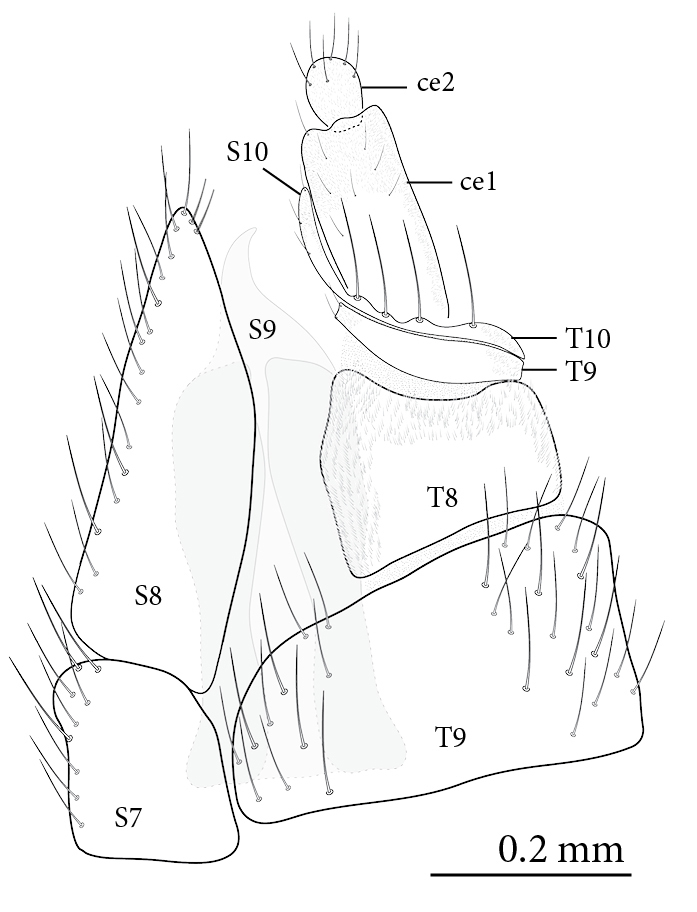
*Neoempheria puncticoxa* Edwards, 1940 adult. Female terminalia; ce1 – first cercomere, ce2 – second cercomere, S7-10 – sternites 7-10, S9 genital fork, T7-10 – tergites 7-10.

**Figure 5.**
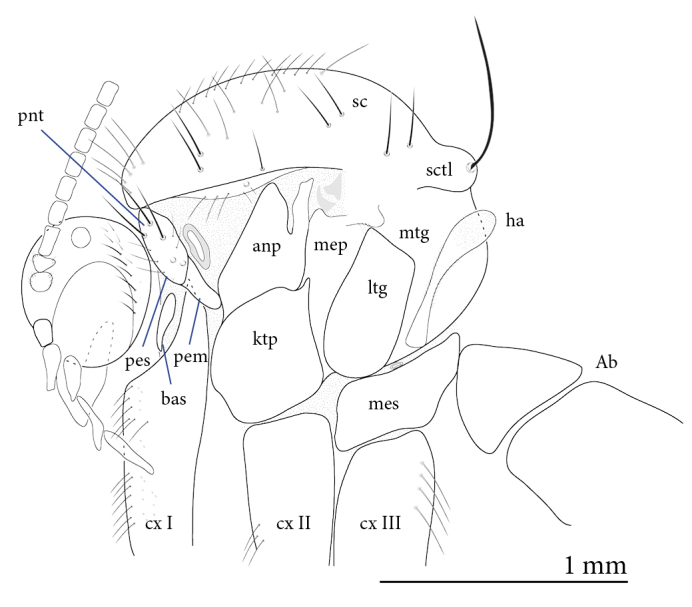
*Neoempheria puncticoxa* Edwards, 1940 adult. Male thorax; Ab – abdomen, anp – anepisternum, bas - basisternum, cxI-III – fore-, mid-, and hindcoxae, ha – haltere, ktp – katepisternum, ltg – laterotergite, anp – anepimeron, mep – mesepimeron, mes – metepisternum, mtg – mediotergite, pem – proepimeron, pes – proepisternum, pnt – pronotum, sc – scutum, sctl – scutellum.

**Figure 6.**
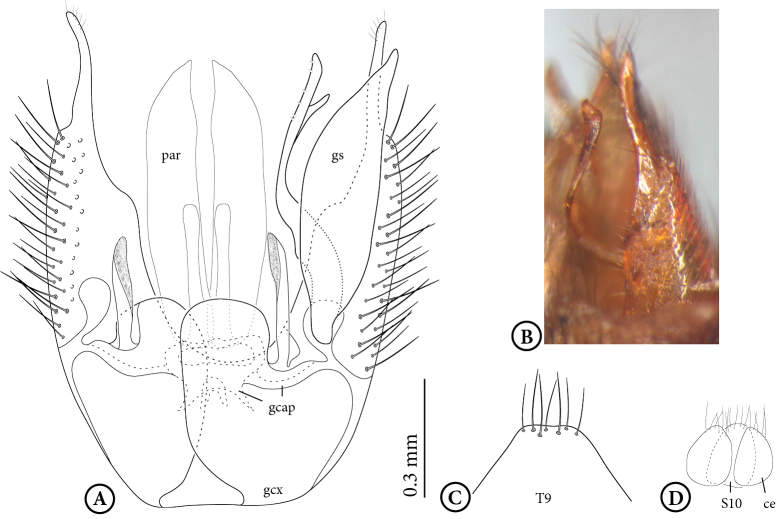
*Neoempheria puncticoxa* Edwards, 1940 adult. Male terminalia, **A** dorsal view; gcap – gonocoxal apodeme, gcx – gonocoxite, gs – gonostylus, par – parameres, **B** lateral view of the gonostylus, **C** Dorsal view of tergite 9, **D** Dorsal view of the cercus and sternite 10.

**Female** (Figs 2, 3b, 4). Similar to male, except as follows: hind coxa with brownish maculae; hind femur browner distally; abdomen yellower (Fig. 2). Wing venation and color pattern as in Fig. 3b. **Terminalia** (Fig. 4). Yellow. Sternite 8 covered with setae, inner margin slightly concave, with a pair of spine-like setae; tergite 8 covered with microtrichia, bare of setae; genital fork well developed, reaching segment 7 anteriorly; cercus short, apical cercomere rounded, ~¼ length of basal cercomere.

**Figure 9.**
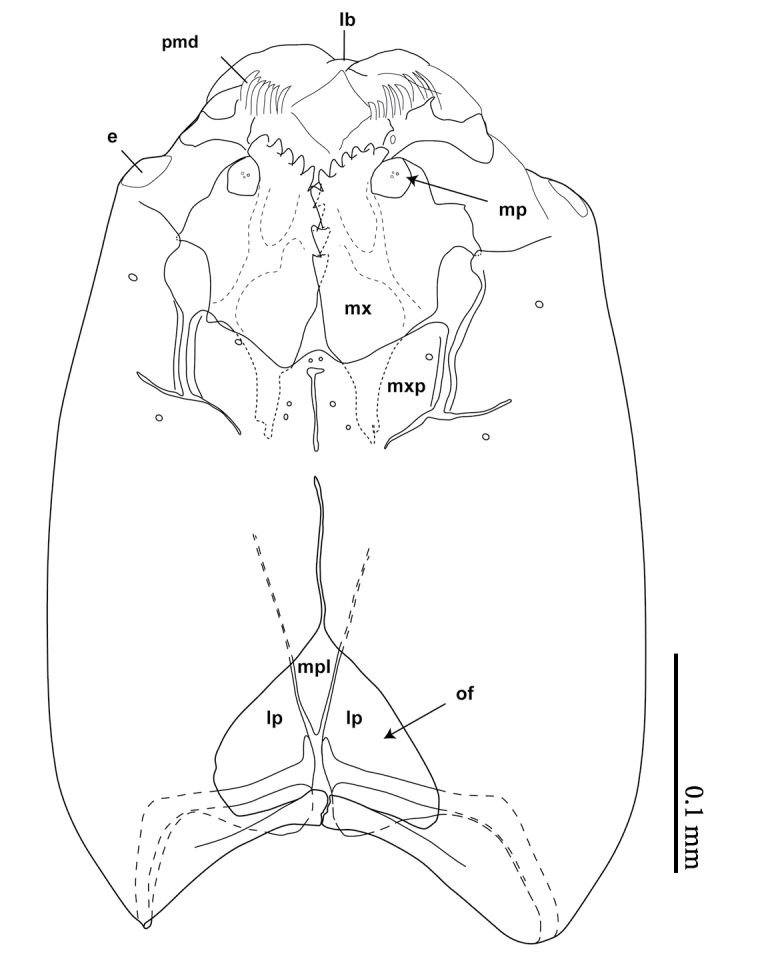
*Neoempheria puncticoxa* Edwards, 1940 larval head capsule. Ventral view, without the mandible; e – eye; lb – labrum; lp – lateral plate; mp – maxillar papilla; mpl – medium plate; mx – maxilla; mxp – maxillary plate; of – occipital foramen; pmd – premandible.

**Mature larva** [Probably fourth instar larva] (Figs 7, 8, 9, 11, 12, 15b). Length: 18.8 mm. General body shape cylindrical, no projections, creamy white in color, whiter in prepupal stage (Fig. 12), 12 apparent segments, segments 4-8 wider and longer than remaining ones.

Head capsule relatively well sclerotized (Figs 7, 8, 9, 12c, d), bare, subrectangular, (anterior end slightly more slender than posterior end, as in the larvae of other mycetophilid genera, e.g., *Brachypeza* Winnertz—see Madwar 1937) and at least partially retractable into first segment. Separation between dorsal plates of head capsule not clearly evident, medial plate extending almost to the posterior capsule margin. Eye posterolateral to the antenna (Figs 7, 9). Occipital foramen ventrally triangular, at about distal fourth of head capsule. Mouthparts occupying ~1/3 of anterior head capsule. Labrum wide, fleshy. Premandible with row of elongated, flexible teeth, supported by a pair of lateral chitinous arms. Mandible semicircular and bearing two rows of medially directed teeth (Fig. 8b) as found in other Mycomyiini species (Krivosheina and Zaitzev 2008). Maxilla with rounded, medially directed, edge bearing a row of medially directed teeth (Figs 8b, 9).

One pair of prothoracic, and seven pairs of abdominal, lateral spiracles; prothoracic spiracle only slightly larger than abdominal ones. Spiracles on short, scale-like sclerite with a couple of small openings. Intersegmental areas with creeping welts (fleshy lobes slightly elongated across the body bearing rows of denticles – Fig. 15b). Each creeping welt includes part of an anterior and a posterior segment, the anterior portion bears fewer, short rows of sparse denticles, the posterior part bears more rows of dense denticles. Posterior end of abdomen with a fleshy lobe folded ventrally.

**Figure 13.**
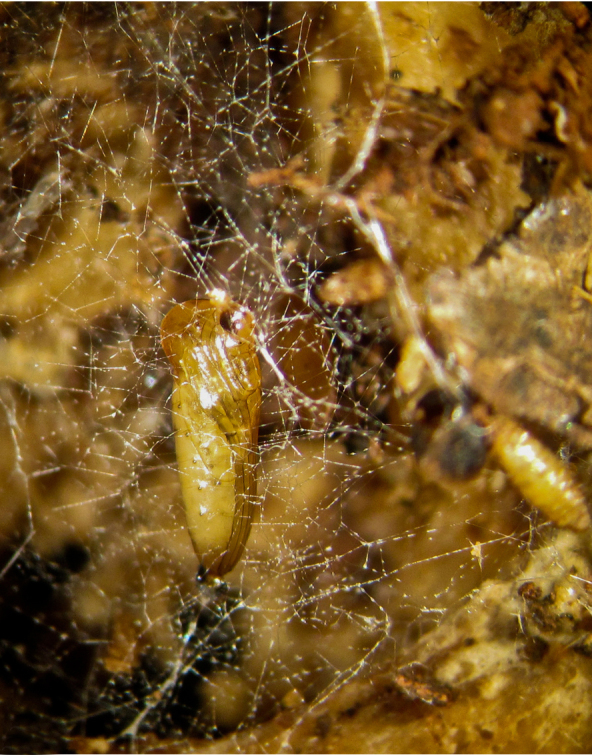
*Neoempheria puncticoxa* Edwards, 1940. Female pupa hanging in its own silk (ventrolateral).

**Pupa** (Figs 13, 14a, 15a). Brown to dark brown, suspended in a web connected by silk lines attached to the entire body, last larval skin remains attached to abdomen posteriorly. Head strongly united with the thorax; developing antennae visible, curved over eyes. Thorax with protruding processes laterally on scutal margin; wing sheath extending just beyond half of abdomen; developing legs held together along ventromedial margin of abdomen. All spiracles flat, flush with surface, not on protrusions. Dorsal margin of abdomen flat.

#### Diagnosis

**Adults.** Laterotergite mostly yellow, anterodorsal margin brownish, bare. Mediotergite yellow ventrally, a brownish triangular mark dorsally, bare. Gonocoxite with large dorsal projection extending beyond apex of gonostylus; gonostylus well developed, deeply bifid, inner branch secondarily bifid and nearly bare, a few small spines on inner margin, external branch strongly setose; parameres strongly developed, with a dorsal, membranous, bare projection. Female apical cercomere rounded, ~¼ length of basal cercomere.

#### Biology

Larvae were found on two different species of polypore fungus, indicating that this species can feed on multiple fungi species, as found in other genera of the family (Hutson et al. 1980, Ševčík 2010). All larvae were collected with the fungus fruiting bodies they were feeding on; those from São Paulo state (all reared material female) were feeding on an uncertain species of *Sparassis* (Polyporales: Sparassidaceae) (Fig. 10a), and those from Goiânia (all reared material male) were collected on an undetermined species of Polyporaceae (Fig. 10b). Both fungi were determined by J.M. Baltazar in vii.2013 using photographs (Fig. 10).

Larvae of *N. puncticoxa* crawled over the surface of the fungal fruiting body (sporocarp), moving along slime trails and silk lines (Fig. 11) they produced. Those on the *Sparassis* sp. often had webs and slime trails suspended between the lamellae of the fungus. The specimens collected on the polypore from Goiânia spent most of their time feeding on the pore surface (underside) of the fungus. It was not observed whether they dig into either sporocarp or not, though there were numerous small holes on the fungus in the region where the larvae were residing. It is probable that they were ingesting the sporulating surfaces of the sporocarp. In preparation for pupation the larvae spun an irregular web, approximating a loosely woven cocoon, and then pupated in the centre of that cocoon, hanging over the substrate with the ventral part of the body facing downwards (Figs 12, 13, 14a). The droplets present on the web strands (Figs 12d, 14a) may be acidic and serve a protective function, as seen in other Sciaroidea (Plachter 1979b, Matile 1997), though this was not tested in our study.

#### Taxon discussion

Edwards (1940) and Coher (1959) both mentioned morphological variation between males and females of *N. puncticoxa*. Coher (1959) highlighted that the pleural marks in females were more pronounced than in the males. We observed the same range of variation in the material we reared (Figs 2, 14b).

## Discussion

### Final remarks

The use of immature data for a phylogenetic reconstruction of the relationships within the Mycetophilidae is still in its infancy, though there is growing information on larvae and pupae of different genera (Krivosheina and Zaitzev 2008, Borkent and Wheeler 2012). There are clear differences between the genera of mycetophilids in terms of larval and pupal morphology, which would provide a novel set of characters for phylogenetic analysis. There are, also differences in biology in terms of fungal taxa on which larvae feed, on how they move and feed on the fungus, how they pupate etc.

With increased information on the natural history of this and related Sciaroidea families, it will be possible to gain a better understanding of the evolution of fungus/Sciaroidea associations, as previously explored for the Keroplatidae (Matile 1997) and for Sciaridae (Shin et al. 2013). Particularly in the Neotropics there is a huge open field in front of us, with information on the biology and natural history of different genera just begging to be discovered, as for putative species of *Mycetophila* Meigen (also reared by SSO), other species of *Neoempheria*, as well as the first association between *Leia* Meigen and its host fungi of the genus *Agaricus* L. (Marco Gottschalk and Felipe B. Valer pers. comm.).

## Supplementary Material

XML Treatment for Neoempheria puncticoxa

## Figures and Tables

**Figure 1a.**
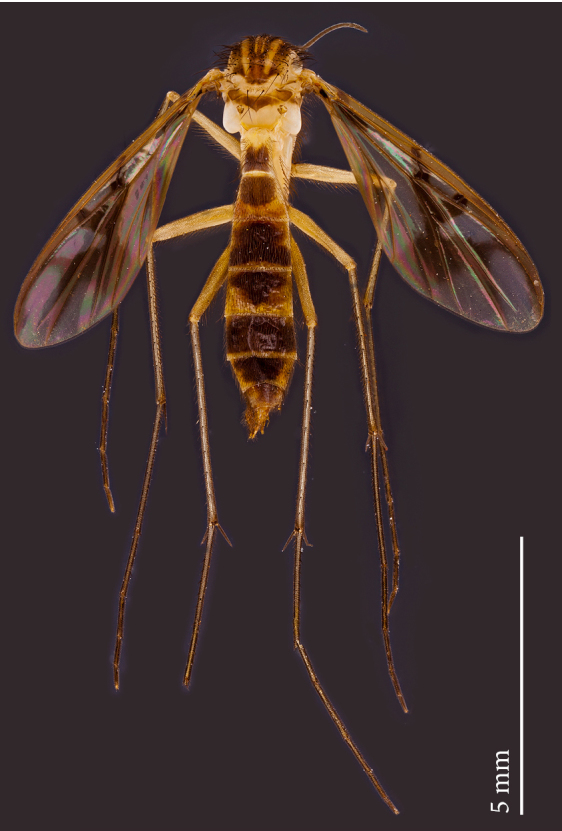
Dorsal view.

**Figure 1b.**
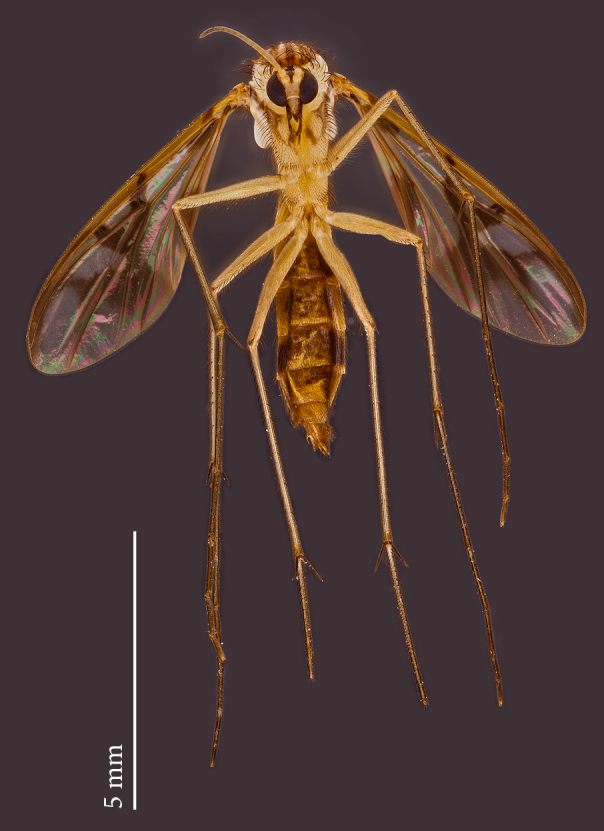
Ventral view.

**Figure 3a.**
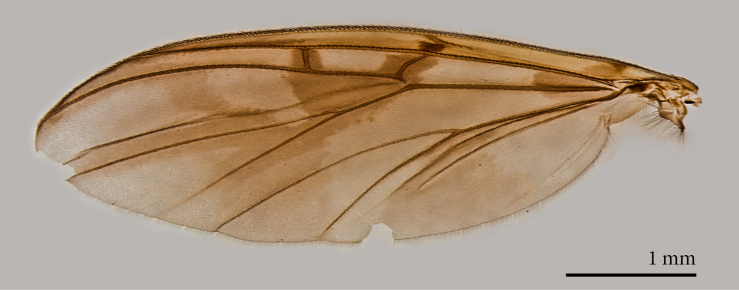
Male.

**Figure 3b.**
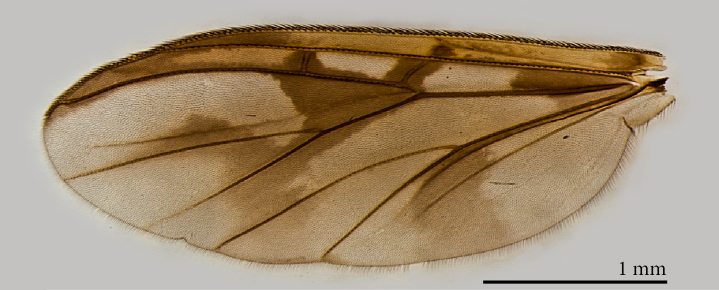
Female.

**Figure 7a.**
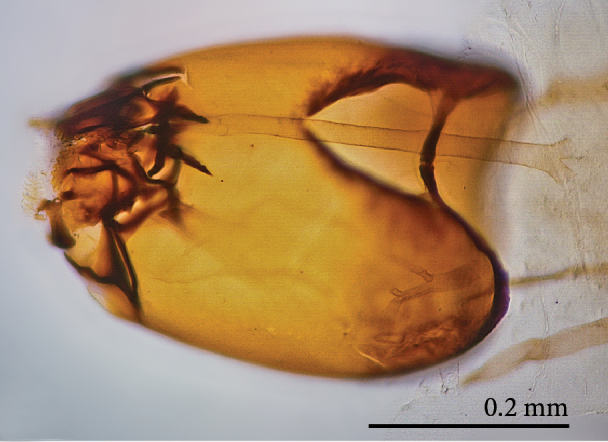
Ventrolateral view.

**Figure 7b.**
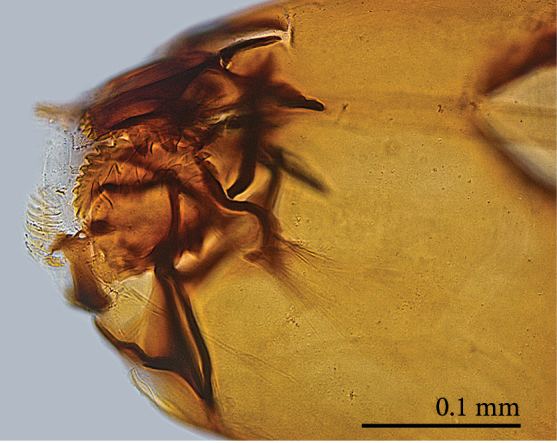
Ventrolateral view.

**Figure 8a.**
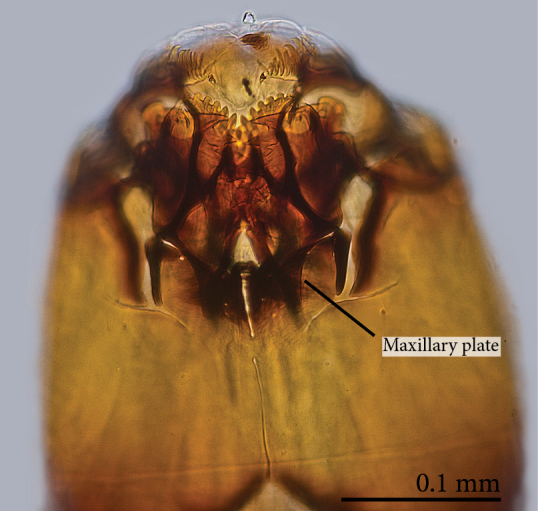
Ventral view.

**Figure 8b.**
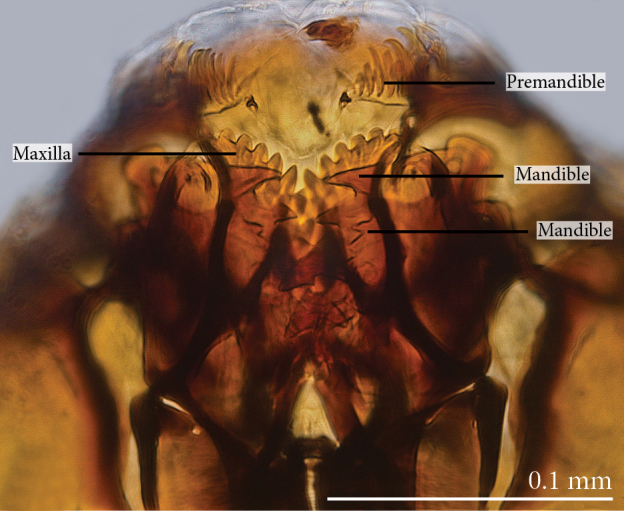
Ventral view.

**Figure 10a.**
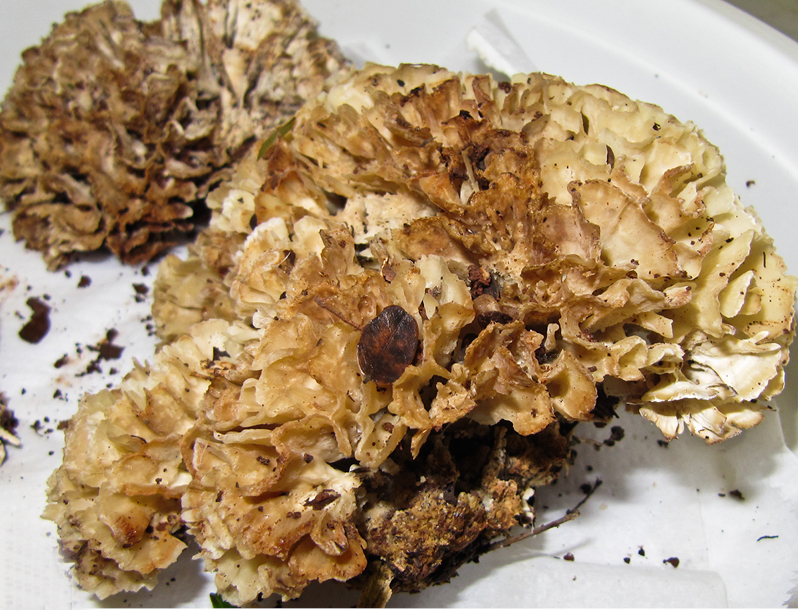
*Sparassis* sp., (Polyporales: Sparassidaceae) fruiting body that larvae of *N. puncticoxa* were found on in São Paulo.

**Figure 10b.**
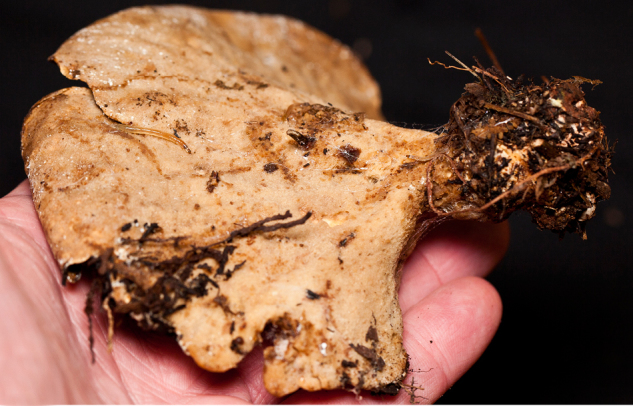
Ventral view of fruiting body of Polyporaceae where larvae of *N. puncticoxa* were found in Goiás.

**Figure 11a.**
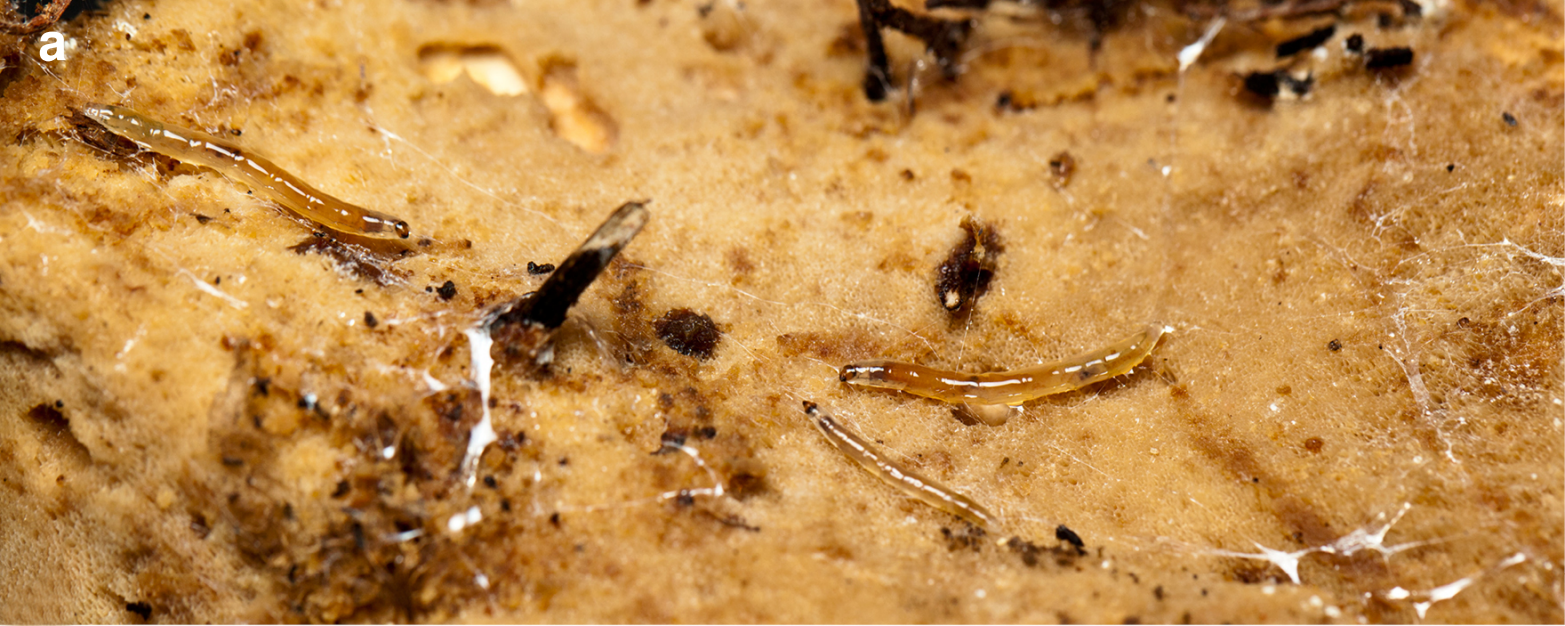
Larvae on ventral face of Goiás fungus, showing webs and slime trails produced by the larvae.

**Figure 11b.**
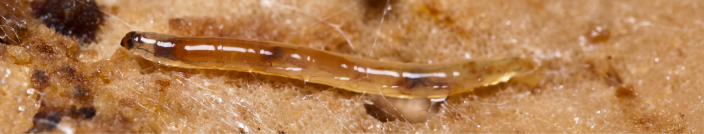
Detail of larvae on ventral face of Goiás fungus, showing webs and slime trails produced by the larvae.

**Figure 12a.**
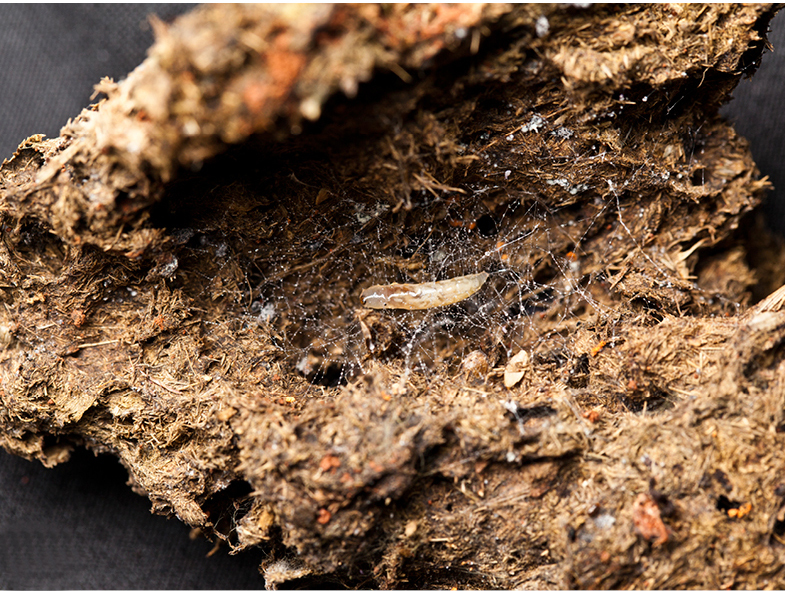
Larva (pre-pupa), ventral view, hanging in its own silk in preparation for pupation. Substrate upside down.

**Figure 12b.**
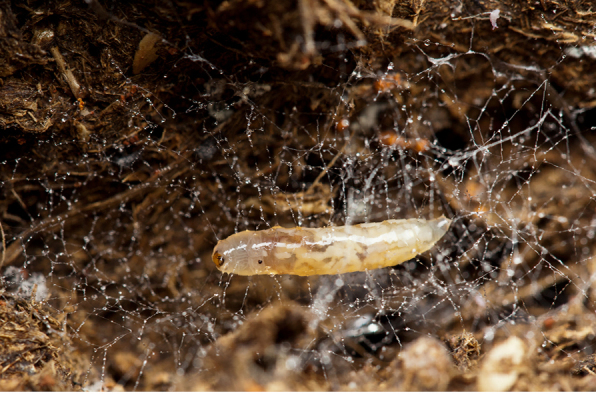
Detail of larva (pre-pupa), ventral view, hanging in its own silk in preparation for pupation. Substrate upside down.

**Figure 12c.**
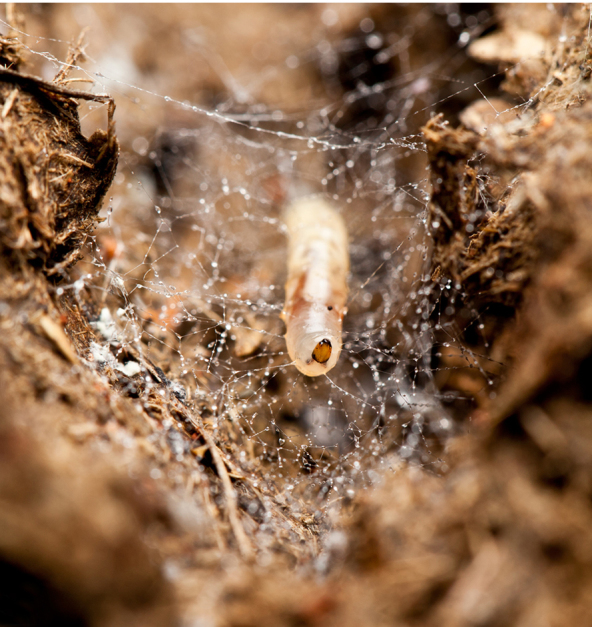
Larva (pre-pupa), frontal view, hanging in its own silk in preparation for pupation. Substrate upside down.

**Figure 12d.**
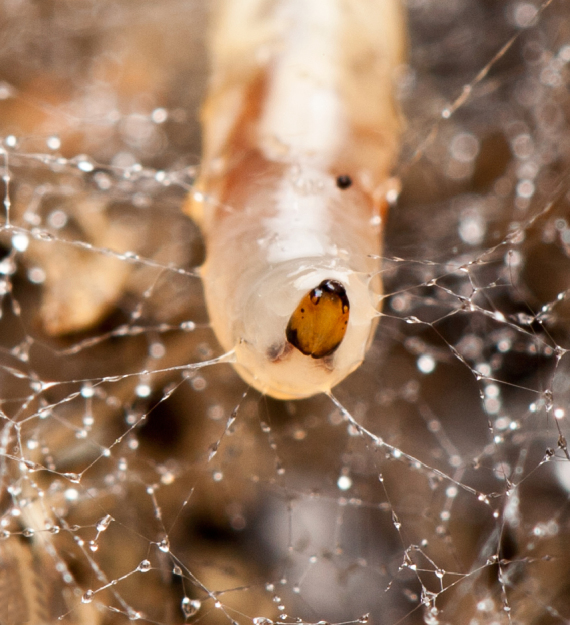
Detail of larva (pre-pupa), frontal view, hanging in its own silk in preparation for pupation.

**Figure 14a.**
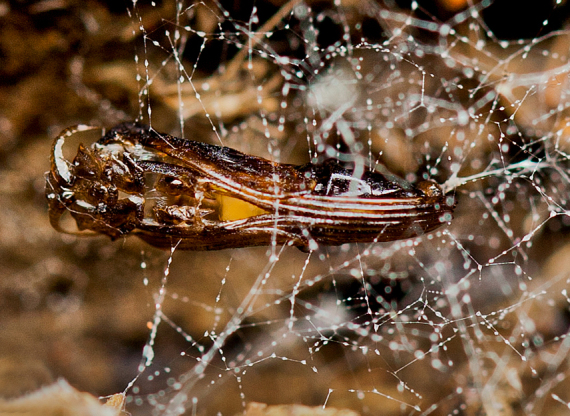
Male pupal exuvia (ventral).

**Figure 14b.**
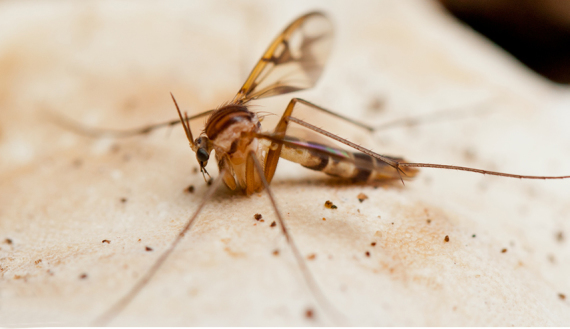
Male imago (dorsolateral).

**Figure 15a.**
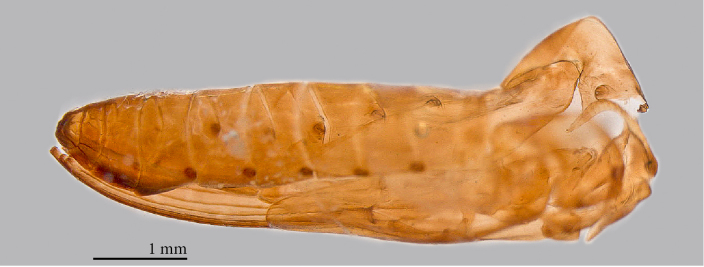
Female pupal exuvia (lateral).

**Figure 15b.**
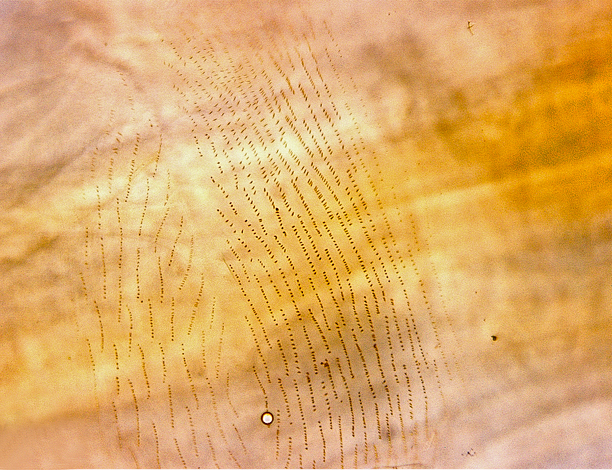
Larva intersegmental areas with creeping welts bearing rows of denticles.
